# An Overview of Current Detection Methods for RNA Methylation

**DOI:** 10.3390/ijms25063098

**Published:** 2024-03-07

**Authors:** Buket Sağlam, Bünyamin Akgül

**Affiliations:** Noncoding RNA Laboratory, Department of Molecular Biology and Genetics, İzmir Institute of Technology, Urla, 35430 İzmir, Turkey; buketsaglam@iyte.edu.tr

**Keywords:** epitranscriptomics, RNA methylation, m^6^A, m^1^A, m^5^C, detection of site-specific methylation, detection of total RNA methylation

## Abstract

Epitranscriptomic mechanisms, which constitute an important layer in post-transcriptional gene regulation, are involved in numerous cellular processes under health and disease such as stem cell development or cancer. Among various such mechanisms, RNA methylation is considered to have vital roles in eukaryotes primarily due to its dynamic and reversible nature. There are numerous RNA methylations that include, but are not limited to, 2’-O-dimethyladenosine (m^6^Am), *N*^7^-methylguanosine (m^7^G), *N*^6^-methyladenosine (m^6^A) and *N*^1^-methyladenosine (m^1^A). These biochemical modifications modulate the fate of RNA by affecting the processes such as translation, target site determination, RNA processing, polyadenylation, splicing, structure, editing and stability. Thus, it is highly important to quantitatively measure the changes in RNA methylation marks to gain insight into cellular processes under health and disease. Although there are complicating challenges in identifying certain methylation marks genome wide, various methods have been developed recently to facilitate the quantitative measurement of methylated RNAs. To this end, the detection methods for RNA methylation can be classified in five categories such as antibody-based, digestion-based, ligation-based, hybridization-based or direct RNA-based methods. In this review, we have aimed to summarize our current understanding of the detection methods for RNA methylation, highlighting their advantages and disadvantages, along with the current challenges in the field.

## 1. Epitranscriptomics

Ribonucleic acid (RNA) serves as an important link between DNA and proteins during transmission of the genetic code based on the central dogma of molecular biology. However, the transmitted message does not always result in a corresponding change in protein abundance, suggesting that post-transcriptional processes contribute to RNA fate as well [1]. In this regard, the fate of RNA may be dictated by epitranscriptomic processes, which are all biochemical modifications in an RNA molecule that impact the fate of RNA without changing the ribonucleotide sequence. To a certain extent, there is an analogy between epigenetics and epitranscriptomics in that neither involves any change in the nucleotide sequence [2]. Such biochemical modifications include pseudouridylation [2], acetylation [3], phosphorylation [4], glycosylation, methylation [2,5] and editing [6]. Recently, epitranscriptomics has drawn a great deal of interest among researchers due to the impactful functional consequences of such modifications [5,7]. In this review, we will solely focus on RNA methylations and refer the readers to excellent reviews on RNA editing and other biochemical modifications [8,9,10].

**Figure 1:**
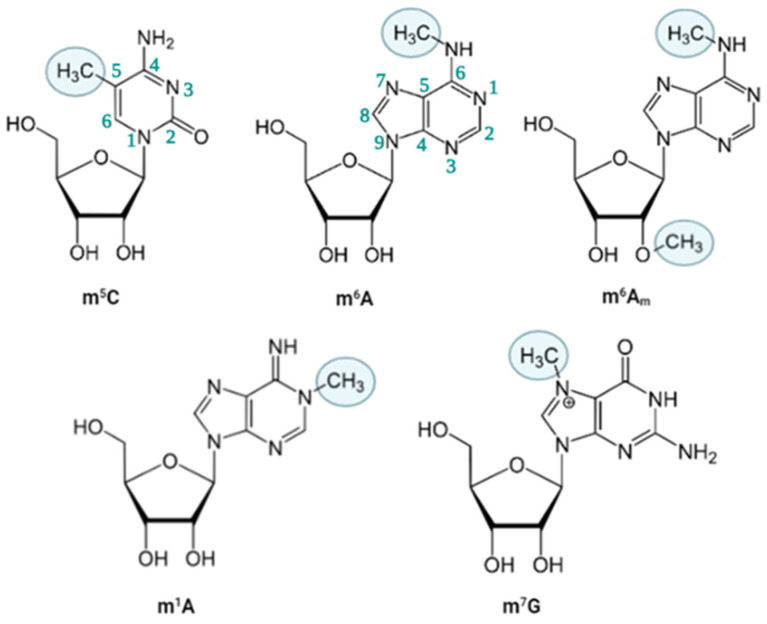
The chemical structures of methylated nucleotides. 5-methylcytosine (m^5^C), *N*^6^-methyladenosine (m^6^A), 2’-O-dimethyladenosine (m^6^Am), *N*^1^-methyladenosine (m^1^A), and *N*^7^-methylguanosine (m^7^G).

Among over RNA 170 modifications, methylation has attracted the vast majority of attention due to its influence on critical cellular processes such as cell cycle and survival [2,11]. Thus far, approximately 10 different methylation marks have been reported on different bases of RNA, all of which are reversibly regulated by various proteins. *N*^6^-methyladenosine (m^6^A), *N*^6^,2′-*O*-dimethyladenosine (m^6^A_m_), *N*^1^-methyladenosine (m^1^A), *N*^7^-methylguanosine (m^7^G) and 5-methylcytosine (m^5^C) represent some of the most common types of methylation [12] (Figure 1). RNA methylation marks have a great impact on RNA stability [13], mRNA translation [14,15], alternative splicing [16], liquid–liquid phase separation [17] and nuclear export of RNA [16,18]. Consequently, these marks are overwhelmingly effective on the cellular fate such as stem cell fate determination and embryonic development [19,20,21]. Any derailment in RNA methylation perturbs the physiological cellular location and fate of transcripts, leading to cell death or diseases [22,23,24,25,26].

## 2. Types of RNA Methylation

A variety of ribonucleotides are subject to methylation. In addition to 2-*O*-methylation, adenosine, guanosine and cytosine nucleotides can be methylated at different sites, the examples of which include, but are not limited to, m^6^A, m^6^A_m_, m^1^A, m^7^G as the cap at the 5’ end and m^5^C [9]. Although methylations on tRNAs and rRNAs have been historically recognized as instrumental biochemical modifications for their functionality, recent reports underline the significance of these marks on mRNAs as well [27]. The deposition, recognition and removal of RNA methylations are performed by specific modifiers, leading to a reversible and dynamic RNA methylation pattern in a cell- and condition-specific manner [28]. The addition of methylation is carried out by methyltransferase enzymes called “writers” while methyl groups are removed by demethylases called “erasers” [27,29]. The destiny of methylated RNA is primarily dictated by an RNA-binding protein (RBP) called “reader”, which recognizes the methylated residue directly or the secondary structure of RNA formed as a consequence of methylation [30]. In the following sections, we will describe the most common types of RNA methylation marks followed by site-specific or genome-wide detection methods. We deeply apologize for being unable to cover all RNA methylations and other excellent studies due to space limitations.

### 2.1. m^6^A

m^6^A is an adenosine nucleotide whose *N*^6^ position is biochemically modified through the attachment of a methyl moiety and ranks as the most abundant modification on mRNAs (Figure 1) [31]. Although mammalian cells were reported to possess m^6^A methylated RNAs as early as 1970s [32], it has taken nearly 30 years to eradicate the doubts about the presence of internal m^6^A residues [33,34]. m^6^A marks have been reported in numerous species such as yeast, *Arabidopsis*, *Drosophila* and mammals [35]. The m^6^A deposition is performed by a writer complex composed of methyltransferase-like protein 3 (METTL3), methyltransferase-like protein 14 (METTL14), Wilms’ tumor 1-associated protein (WTAP), RNA-binding motif protein 15 (RBM15) and other methyl-transferase proteins. The actual catalysis is carried out by METTL3, and METTL14 functions as an allosteric activator for METTL3 stability [36,37]. METTL3 and METTL14 are responsible for 99% of m^6^A deposition on mRNAs [38]. All other writer proteins are also involved in facilitative tasks for m^6^A addition, such as localization and stability on target RNA site [39,40,41]. In human and mouse, m^6^A-specific RNA immunoprecipitation studies coupled with high throughput sequencing studies have identified the consensus motif RR(m^6^A)CH in which R represents A or G, and H represents A, C or U [42,43]. Minor variations have been reported in other species. Additionally, a second motif, m^6^ACA, has been reported, which is sensitive to digestion by the MazF RNAse if the adenosine is methylated [44]. This feature has been exploited to study m^6^A RNA methylations in a site-specific or genome-wide manner. Thus far, merely two eraser proteins have been identified that serve as m^6^A demethylases, namely fat mass and obesity-associated protein (FTO) and alpha-ketoglutarate-dependent dioxygenase homolog (ALKBH) [18,45].

m^6^A methylated RNAs are recognized by a class of RNA-binding proteins, readers, that can be divided into two groups based on their mode of recognition: (1) direct readers recognize the methyl moiety to coordinate the downstream events; (2) indirect readers recognize and fine-tune the secondary structure of the methylated RNA to modulate the interaction between the methylated RNA and other proteins. Direct readers include YTH domain-containing proteins (YTHDC), YTH domain-containing family proteins (YTHDF) and eukaryotic initiation factor 3 (elF3) [46,47]. Among indirect readers are heterogenous nuclear ribonucleoprotein C (HNRNPC), heterogenous nuclear ribonucleoprotein G (HNRNPG), insulin-like growth factor 2 mRNA-binding protein (IGF2BP) and Fragile X mental retardation protein (FMRP) [48]. Readers can be located either in the cytoplasm or nucleus to perform m^6^A-mediated cellular processes. In the nucleus, YTHDC1 modulates splicing and transport of mRNA into the cytoplasm, where YTHDC2 dictates the post-transcriptional fate of cytoplasmic mRNAs by regulating their stability and translational efficiency. Both readers recognize the m^6^A portion on RNAs [48,49]. Indirect readers HNRNPC and HNRNPG proteins modulate splicing by interacting with noncoding RNAs such as miRNA (microRNA), snRNA (small nuclear RNA), snoRNA (small nucleolar RNA) [50,51,52] while IGF2BP1–3 proteins bind weakly to the m^6^A moiety, supporting the stability of the methyl-adenine interaction. In addition, FMRP interacts with m^6^A-bound YTHDF2 and indirectly facilitates m^6^A stability [53].

### 2.2. m^6^A_m_

*N^6^*, 2′-O-dimethyladenosine, often abbreviated as m^6^Am, is a type of methylation that carries two methyl moieties, one at the *N^6^* position of the adenine ring and another at the 2’ position of the ribose sugar. This m^6^A_m_ modification influences the fate of RNAs through splicing, stability and translation. It also has implications in transcriptional processes through the modulation of chromatin interactions [54,55]. Therefore, it has been implicated in several developmental disorders as well as diseases such as cancer, obesity and neurodegenerative diseases [56,57,58].

Unlike other methylation marks, m^6^A_m_ does not have a distinct sequence motif on RNA. However, the BCA motif, where B represents C, U or G, has been explored as being part of the RR(m^6^A)CH consensus motif at the 5’ cap structure [59,60]. Additionally, m^6^A_m_ can be deposited at the 5′ cap structure, 7-methylguanosine (m^7^G), that can be further modified with the aid of cap-specific adenosine-N^6^-MTase (CAPAM) protein, also called Phosphorylated CTD-Interacting Factor 1 (PCIF1) to m^6^A_m_. In this case, the methyl group is deposited to the *N^6^* position of the adenine base. Thus far, PCIF1 protein has been reported as the sole writer protein for m^6^A_m_ addition [61]. On the other hand, FTO demethylase catalyzes the removal of m^6^A_m_ modification. AlkB family member 5 (ALKBH5) is incapable of removing m^6^A_m_ marks [62,63].

### 2.3. m^1^A

m^1^A carries a methyl moiety on a nitrogen attached to the first carbon atom of adenosine (Figure 1) [64]. Its stoichiometry is very low compared to the m^6^A methylation due to its location on adenosine. m^1^A differs from m^6^A in that m^1^A generates a positively charged nucleotide due to its location, the Watson–Crick interface [65]. Consequently, the resulting positive charge modulates the nature of RNA-protein interactions as well as the RNA secondary structure. Thus far, more than 2500 m^1^A sites have been identified in human despite the fact that more attention has been directed towards RNA m^6^A methylation [66]. m^1^A marks are generally located in the 5′ untranslated region (5′ UTR) of mRNAs, but mostly in rRNA and tRNA of eukaryotes, as well as mitochondrial RNA [67].

The writer complex of m^1^A methylation is composed of tRNA methyltransferases located in the cytoplasm, namely TRM61, TRMT6, TRMT10C and TRMT61B [68]. The TRMT6-TRMT61A complex utilizes the GUUCRA consensus motif for addition of m^1^A [69,70]. AlkB homolog 1, histone H2A dioxygenase or alpha-ketoglutarate-dependent dioxygenase ABH1 (ALKBH1) and AlkB homolog 3, alpha-ketoglutarate-dependent dioxygenase (ALKBH3) serve as eraser proteins of m^1^A [67]. In addition, FTO has been reported to remove m^1^A marks on tRNA [71]. m^1^A reader proteins include YTHDF1, YTHDF2, YTHDF3 and YTHDC1. It is worth noting that the number of m^1^A marks on mRNA and noncoding RNAs (ncRNAs) is relatively scarce [67,72,73].

### 2.4. m^7^G

m^7^G is another modification of guanosine nucleoside located at the seventh nitrogen where the 5′ cap of RNA is established. This methylation is involved in mRNA capping, which has crucial roles in mRNA stabilization, translation initiation, nuclear export and internal modifications in other types of RNAs such as rRNA, tRNA, and some miRNAs [74]. The addition of m^7^G modification is facilitated co-transcriptionally by guanylyl-transferase methyltransferase (GTase). On mRNAs, the methylase adds the m^7^G at the 5′ cap, whereas the deposition site on tRNAs is characterized by a consensus sequence “RGGUY’’. The writer protein complex of m^7^G for tRNA is composed of methyltransferase 1, also called tRNA (guanine-N(7)-)-methyltransferase (METTL1), and tRNA (guanine-N(7)-)-methyltransferase subunit WDR4 (WDR4) writer proteins in mammals and their orthologs Trm8/Trm82 complex in yeast. Additionally, the RNA guanine-7 methyltransferase and RNMT-activating mini protein (RNMT-RAM) complex functions in the deposition of m^7^G at the mRNA caps by transferring a methyl group from S-adenosylmethionine (SAM). Additional regulators have been reported in m^7^G modification of rRNAs [75]. Quaking (QKI) protein has been reported as the first reader protein of m^7^G, including three isoforms, QKI5, QKI6 and QKI7 [76]. However, no demethylases for m^7^G have been reported yet. It is important to note that this modification has crucial roles in various diseases such as genetic disorders, cancer and viral infectious [77,78,79].

### 2.5. m^5^C

m^5^C is a type of methylation in which a methyl moiety is covalently attached to the fifth carbon of cytosine (Figure 1). The existence of m^5^C in RNAs was reported nearly 50 years ago [32]. However, exploiting bisulfite treatment that is typically used to examine DNA methylation, m^5^C marks were shown to exist internally in mRNAs and lncRNAs as well [80]. The components of m^5^C biogenesis have been linked to various diseases. For instance, m^5^C writer protein NOP2/Sun RNA methyltransferase 2 (NSUN2) has been reported to promote tumor progression [81]. most m^5^C sites on RNAs are recognized by proteins that possess an S-adenosyl methionine (AdoMet)-binding region, a prevalent catalytic domain [81,82,83,84,85,86]. Thus far, tRNA methyltransferase 1 (TRDMT1), also known as DNA methyltransferase homolog DNMT2, has been shown to be a m^5^C methyltransferase just like NSUN2 [87]. Recently, NSUN1 and NSUN3–7 proteins have also been sifted out as m^5^C writer proteins [86,88,89,90]. Although an exact consensus sequence for m^5^C is still uncertain, a few potential sequences, namely, HACCR, CWUCUUC and CCDCCR, have been reported in *Arabidopsis thaliana* [91]. Erasers of m^5^C include TET family of enzymes and ALKBH1 [92,93,94]. Lastly, two reader proteins have been reported, namely Aly/REF export factor (ALYREF), an oncogenic factor, and Y-box-binding protein 1 (YBX1) [95,96]. Compared to m^1^A and m^6^A marks, m^5^C marks have been characterized relatively poorly. Thus, more work is required to elucidate the contribution of m^5^C marks in health and disease [86,94].

## 3. Detection Technologies for RNA Methylation

**Figure 2:**
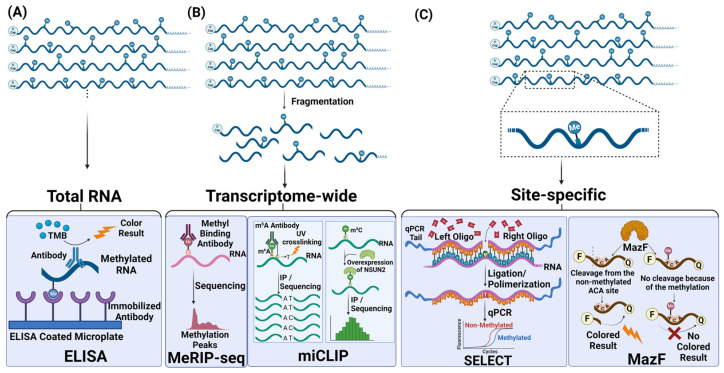
Different approaches for mapping methylation marks. (**A**) The approaches for detection of total RNA methylation abundance by ELISA with specialized antibody binding and colorimetric measurements (**B**) Transcriptome-wide mapping analysis methods for methylation-site detections by using fragmentation and further immunoprecipitation techniques; MeRIP-seq and miCLIP (**C**) Site-specific abundance of methylation detection approaches for desired methylation types based on ligation and cleavage of their specialized motifs by methods of SELECT and MazF, respectively. Created with BioRender.com.

Various methods have been developed to evaluate the extent of RNA methylation (Figure 2). The method of choice is primarily dictated by the coverage and resolution desired in addition to cost, simplicity and expertise required (Table 1). Perhaps, the initial choice in most studies is the global assessment of fluctuations in total RNA methylation upon a stimulus. Since the global assessment lacks resolution, approaches have been developed to examine the methylation status of specific sites, especially if existing data point to the significance of a specific site. On the other hand, genome-wide approaches are exploited to cover the whole transcriptome in an unbiased manner. As such, these methods involve the use of methylation-specific antibodies, digestion of specific sequences, ligation of methylated sites, hybridization of corresponding RNAs or labelling. In addition to the coverage and resolution, cost, efficiency and the required technical infrastructure are other criteria that dictate the type of detection method to be used.

### 3.1. Approaches to Measure Global Changes in RNA Methylation

Global detection methods refer to the overall measurement of methylation, either total methylation or a specific type of methylation. One application of this approach is to measure the amount of total methylation without considering the transcripts carrying it or the site of methylation. This type of analysis facilitates the direct detection of RNA methylation status using total RNA isolated from any species or cells under a certain cellular condition. Typically, this approach is used to establish a link between the change in methylation abundance and a phenotype of interest. Enzyme-linked immunosorbent assays (ELISA)-based colorimetric kits are commercially available. Basically, the intensity of the signal originating from the sample RNAs is compared to a standard curve obtained from known methylated and nonmethylated control RNAs to assess the extent of methylation in test samples [97]. The major drawback of this approach is that it is impossible to deduce which transcripts or which nucleotide residues are affected from differential methylation.

Mass spectroscopy (MS) is another method utilized to analyze the total amount of RNA methylation based on their polarity and charge. This technique involves enzymatic digestion of RNAs into nucleosides, followed by mass spectrometry analysis to identify modified nucleosides. Based on the mass-to-charge ratio the identification and quantification of individual molecules are determined. Perhaps the most classical method for the analysis of modified RNAs is one-dimensional (1D) or two-dimensional (2D) thin layer chromatography (TLC). This approach takes advantage of modified nucleotides having a different net charge, hydrophobicity or polarity compared to their nonmodified counterparts. Particularly, 5′- or 3′-nucleoside monophosphates can be analyzed using this relatively simple system [98]. The comparative quantification of methylation is put into practice by forward methods such as liquid chromatography–tandem mass spectrometry (LC-MS/MS) or 2D-TLC [99,100,101]. LC/MS methodology requires cleavage of a single nucleotide with the aid of RNases and UV detection of desired methylation by using its physico-chemical properties. Although this quantitative method has straightforward steps, the localization or sequence information cannot be derived and any contamination by foreign RNA artifacts can affect the downstream analysis [99,100]. Aforementioned 2D-TLC method is a deep-rooted approach for separation of molecules in a sample depending on its size and charge. To be able to tune this method to methylation detection, cellulose substrate is used to disperse RNA based on properties changed by the effect of methylation: charge and hydrophobicity. The fragmentation of RNA is performed based on the methylation type of interest [102]. For example, RNase T1, which recognizes GAC motifs, is utilized for detection of m^6^A and normalized to the total adenosine level. The observation is performed with ultraviolet light or by γ-32P-ATP radioisotopes for 5′-end labeling to increase the sensitivity. Albeit with high accuracy, 2D-TLC can only detect m^6^A on GAC context but cannot catch AAC sites present in rRNAs [98,101,102].

A combined approach, site-specific cleavage and radioactive labelling followed by ligation-assisted extraction and TLC (SCARLET), exploits RNase H site-specific cleavage if there is a putative target methylation site. After the site-specific cleavage, splinted ligation is utilized to ligate the corresponding nucleotide to a DNA oligo, preventing it from digestion by RNase T1/A. This process is followed by thin-layer chromatography for evaluation of m^6^A [103,104].

Dot blot, also called slot blot, is a relatively basic method to examine different types of RNA methylations. It is performed by using a membrane coated with specific antibodies or molecules that recognize the desired methylation. The detected methylations are spotted on the membrane by signals performed with the aid of a fluorescence or chemiluminescence molecule after subjecting a vacuum process [105]. Although it is often used for m^6^A, it can be modified to entertain the detection of other types of methylations such as ^5^hmC [106]. As a negative side of this technique, it can only be used to verify the presence of methylation and to compare the changes in the global abundance of methylation among different samples. The dot blot approach is incapable of pinpointing the precise site of methylation mark. Of importance is the urgency to eliminate potential DNA contamination while analyzing ^5^hmC marks on RNA [105,107].

As a novel technique to identify and quantify the genome-wide methylation in RNA, DART-Seq (Deamination adjacent to RNA Modification Targets) was improved at the single-nucleotide level. This method is an antibody-free approach for m^6^A detection of RNA taking advantage of apolipoprotein B mRNA editing enzyme catalytic subunit 1 (APOBEC1). The method involves five steps, namely RNA isolation, in vitro deamination, library preparation, deep sequencing and data analysis. The in vitro deamination step involves APOBEC1, a chimeric protein engineered by fusing the m^6^A-binding YTH domain, which deaminates the cytosine nucleotide adjacent to m^6^A to uracil. This targeted deamination strategy offers a high specificity for m^6^A sites. In the last step, the abundance of reads stemming from unconverted cytosine nucleotides reflects the level of m^6^A modification at that specific site. DART-Seq method can be employed to quantitatively measure the extent of m^6^A RNA methylation in a transcriptome by coupling the analysis with RNA-seq [108].

### 3.2. Transcriptomic Detection Methods

Most genome-wide approaches in current use employ second generation sequencing (NGS) in which an amplification step is required to generate a cluster of templates for detectable sequencing signals in an unbiased manner. With a low error rate, NGS has been the method of choice despite its shorter read of a couple of hundreds of nucleotides at the most. Single-molecule direct sequencing protocols have been developed to overcome the size limitation. Currently, there are two direct RNA-based detection methods, namely nanopore and single-molecule real-time (SMRT) applications [35,109,110]. Initially, nanopore technology employed a technique for determination of characteristic current blockade difference based on DNA and RNA structure changes while passing through nanopores. This method was applied to the analysis of m^6^A and m^5^C marks. These modifications have been tested by sequencing of methylated and nonmethylated synthetic RNAs. The resulting difference is calculated and used to locate the methylated region of RNA [109]. The other approach, SMRT, takes advantage of labelled nucleotides during SMRT DNA library preparation followed by LC-MS observation. Although this procedure assures determination of methylation-related isoform or transcript changes and the quantity of methylation sites per isoform, it is not a sensitive enough protocol as the margin of error is relatively much higher [7,110,111].

The genome-wide analysis involves the sequencing of the precipitated RNAs, whereas qPCR can be employed, with a proper set of primers, to examine the precipitation efficiency, thus the existence of a mark, of a single target RNA. For instance, m^6^A-seq/MeRIP-seq is an RNA immunoprecipitation method that involves the use of an m^6^A-specific antibody and RNAs fragmented into approximately 100 nt in size [34]. The sequencing of precipitated RNA provides valuable information about the enrichment of a fragment of RNA as an indicator of the presence of a methyl moiety. Alternatively, a pair of primer can be designed for qPCR analysis of a target RNA to check for its enrichment in the immuno-precipitate. To compare the relative change in the extent of methylation upon a treatment (for example, control versus drug treatment or healthy versus cancer), RNAs in each condition must be sequenced first to determine the abundance of individual RNAs as treatments may lead to an increase in the RNA abundance independent from differential methylation. Subsequently, the relative enrichment of target RNAs or fragments of target RNAs must be calculated to find out the fold of differential RNA methylation. There are various library preparation and sequencing strategies for detection of modified nucleotides in RNAs [112]. MeRIP-seq is a primary choice of method since it can be adapted to examine any modification as long as a specific antibody is available [33,34]. However, cross-reactivity among antibodies is an important issue that should be always taken into consideration in antibody-based approaches as cross-reactivity may lead to false-positive signals. For example, it is highly challenging to distinguish m^6^A from m^6^Am due to nonspecific interactions between antibodies and methylation marks. Although adaptability to the analysis of different methylation marks makes MeRIP-seq an attractive choice, a major drawback of this method is its low resolution. Post-precipitation, RNA fragments of 100–200 nt in size are subjected to sequencing, making it difficult to pinpoint the exact site of methylation especially if multiple methylation motifs exist in such fragments [33,34,111].

m^6^A individual-nucleotide resolution UV crosslinking and immunoprecipitation (miCLIP) [59] and its improved version, enhanced CLIP (eCLIP) [113], is another antibody-based approach to map m^6^A sites at the single-nucleotide resolution. This approach identifies m^6^A marks with high confidence as the UV-crosslinked nucleotide nearby the methyl mark serves as an excellent indicator for the presence of a methyl moiety. UV crosslinking leads to a C-to-T mutation signature that ensures the partition of multiple m^6^A signals within the same peak. More importantly, this approach makes it possible to distinguish m^6^A from m^6^Am. Although miCLIP can overcome all the drawbacks of MeRIP-seq, it requires a higher quantity of input material and cannot provide stochiometric information [111,114,115].

MeRIP and miCLIP can be utilized for both transcriptome-wide and site-specific mapping of m^6^A, m^1^A, m^6^Am and m^5^C marks. Dynamic changes in the abundance of these types of RNA methylation marks can be examined by exploiting relatively strong interactions between specific methylation marks and antibodies that specifically recognize these marks [35]. Following the incubation of RNA samples with antibodies, the fragments of methylated RNAs are immune-precipitated with an antibody. Additionally, the comparison of the relative amount of site-specific methylation changes can be observed following site-directed mutagenesis or RT-PCR (real-time PCR) reaction by specific primers after immunoprecipitation [116].

**Table 1:** The summary of commonly used methylation detection methods.

Methods	Advantages	Disadvantages	References
**Global RNA Methylation Detection Methods**			
ELISA	Various methylation detection based on antibody presence Low amount of input RNA requirement Standard curve convenience Easy preparation steps with commercial product	Contamination risk during preparation Transport and storage conditions of commercial kit require attention Instability of RNA and antibodies provided by commercial kit require attention Long standby times during protocol and labor intensive	[97]
2D-TLC	Conventional method Increased accuracy by radioisotopes Increased specificity of m^6^A in mRNA by cleavage of GAC context Low amount of input RNA required High stoichiometric information Adaptable to all types of modifications	Inability to detect AAC sites of m^6^A in rRNA Difficulty of using radioactive substance Low resolution	[98,101,102]
LC-MS	Standardized technique High accuracy in quantification Easy to prepare Useful for all types of modifications	Risk of contamination No sequence information Labor intensive Necessity for specialized equipment Require complicated computational analysis	[99,100]
SCARLET	High accuracy Site-specific determination Low amount of input RNA required No need for specialized equipment	Only one site per transcript determination at once Low throughput High quantity of input material Used for only m^6^A methylation	[103,104]
Dot Blot	Simple Relatively inexpensive No requirement for fragmentation Markedly saves time since no need chromatography, gel electrophoresis or complex gel blocking procedures	Need to use antibody or other molecule for fluorescence or chemiluminescence Cannot determine the quantitation Unable to determine precise location	[105,106,107]
DART-Seq	High sensitivity for m^6^A sites at the single-nucleotide level An antibody-free approach to eliminate commercial assays Low input RNA requirement with as little as 10 ng of total RNA as input High-throughput sequencing Long-read compatibility	Potential off-target deamination due to specificity of APOBEC1 Requires careful consideration during data analysis due to specificity of APOBEC1 Computational challenges as robust computational pipelines for accuracy	[108]
**Transcriptome-wide RNA Methylation Detection Methods**			
Nanopore	Possibility of methylation-related isoforms and transcripts examination Validation of potential methylation stoichiometry Facility in abundance of methylation per isoform determination Library preparation is not required No need for PCR or qPCR equipment	Newly adopted to methylation detections Depend on change in the current which can be hard to differentiate Fewer studies with RNA isolated from cells High level error rate in base assignment Prone to statistical problems	[109]
SMRT	Possibility of methylation-related isoforms and transcripts examination Detection methylation level per isoform	Low level of sensitivity High level of error in base assignment Prone to statistical problems	[110,111]
MeRIP-seq	Transcriptome-wide mapping is provided Low quantity of RNA input is required Easy steps for library construction Adaption to various methylations (m^6^A, m^1^A and m^5^C) based on antibody availability Well-studied	Necessity of RNA sequencing Inability in discrimination of m^6^A from m^6^A_m_ Insufficient single-nucleotide resolution Insufficient to distinguish multiple methylation sites in a peak Stoichiometric information not provided	[33,34,112]
miCLIP	Transcriptome-wide mapping is provided Increased specificity by a C-to-T mutation signature for m^6^A and NSUN2 overexpression for m^5^C Ability in discrimination of m^6^A from m^6^A_m_ Sufficient to single-nucleotide resolution Sufficient for distinguish multiple methylation sites in a peak Adaptable to all types of methylations	Complicated steps for RNA library construction Stoichiometric information not provided High abundance of input material is needed Requirement to special equipment	[59,113]
**Site-specific RNA Methylation Detection Methods**			
Reverse transcriptase based-qPCR assay	Specific-site detection using related oligomers Stoichiometric approach by melting properties Useful for site-specific detection in rRNA and snRNA as well as mRNA Useful for various modifications Low RNA input required Straightforward method	Low sensitivity level Based on reverse-transcriptase enzyme flexibility	[117,118,119]
HRM	Simple Specific location of m^6^A modification residues with high-throughput measurement Commercially available kits	Necessity of positive and negative control to evaluate methylation abundance Relatively detection of methylation in percentage	[120,121]
MazF	Site-specific determination of m^6^A using ACA sequence cleavage by MazF enzyme Transcriptome-wide mapping available with further processes (MAZTER-seq)	Detection of m^6^A profile only in ACA content Insufficient to distinguish adjacent ACA sites in MAZTER-seq	[122]
T3/T4 DNA ligase-qPCR	Methylation stoichiometry can be observed Site-specific detection Easy protocol steps	Efficiency of the ligase is crucial Low throughput	[123]
SELECT	Feasibility of evaluation of methylation stoichiometry Provided site-specific detection Easy to prepare Able to adapt for various types of methylations (m^1^A, A_m_ (2′-O methyladenosine))	Based on two different enzyme efficiencies: Bst polymerase and Splint R ligase Possibility of false-positive outcomes Rough process of oligomer designing Low throughput	[124]

### 3.3. Site-Specific Detection Methods

Although transcriptomic approaches provide valuable information about changes in methylation marks, its cost, requirement for sophisticated devices and expertise and potential cross-reactivity of antibodies necessitate the use of site-specific detection methods for validation of transcriptomic data. Typically, pre-existing data, such as those obtained from a transcriptomic study, is used to select a site for examination. The existing site-specific methylation detection methods employ hybridization-based, digestion-based and ligation-based approaches.

Hybridization-based approaches take advantage of the incredible mechanism of retrotranscription by nucleic acid polymerases *Thermus thermophiles* (*Tth*) and *Bacillus stearothermophilus* (*BstI*) [117]. The template RNA is retrotranscribed by these polymerases with the aid of an adjacent primer, leading to RNA-directed DNA synthesis. The key point here is the flexibility of polymerases during the elongation process and constructional access of the enzyme to the template with the primer. In principle, methylated nucleotides would impede polymerization. Therefore, RNA templates with methylation would be amplified less efficiently compared to nonmethylated RNA templates. Furthermore, polyacrylamide gel electrophoresis or RT-qPCR is performed to determine the relative level of methylation based on the selective processivity of the enzyme. It is important to note that these approaches were primarily developed to examine m^6^A marks [117,118,119]. Hybridization-based methods provide relative information about the methylation stoichiometry at specific RNA sites and can be used for rRNA and snRNA specific sites. However, this approach suffers from low sensitivity [111]. High-Resolution Melting (HRM), an alternative hybridization-based approach, has been improved to detect m^6^A methylations in a site-specific manner. This method exploits the plots of purely methylated and un-methylated RNA sequences of interest. The RNA region of interest is then compared to these melting curves and gives the result in percentage in a high-throughput manner. Methylation causes a change in the melting curves of 100% methylated and 100% unmethylated RNAs. Therefore, it is a necessity to determine the methylated region on the RNA for processing the melting curve standards [120,121].

A unique methodology of digestion-based method involves the MazF RNA for site-specific detection of methylation marks. MazF is an RNase that digests the nonmethylated ACA motifs and these fragments can be aligned to the genome to uncover the site of methylation. Single-base resolution can be accomplished by MazF by taking advantage of Förster resonance energy transfer (FRET) technique as a fluorescent biosensor of m^6^A by a light production after cutting ACA motif and transfer of energy from the reporter to the quencher in the absence of methylation [44,101,122].

Ligation-based detection methods have been initially utilized to analyze m^6^A marks by comparing the ligation efficiency of two primers complementary to the sequences upstream and downstream from the methylated nucleotides. Liu et al. employed a T3 DNA ligase-based qPCR technique to examine m^6^A marks in a specific region in RNA [123]. Subsequently, Xiao et al. improved this approach by using a single-base elongation- and ligation-based qPCR amplification method (SELECT), in which T3 DNA ligase was replaced by Bst 2.0 DNA polymerase and SplintR ligase [124]. In this technique, two probes, namely forward and reverse oligomers, are designed immediately upstream and downstream from the methylated sites. Two additional oligomers are designed +2/−6 nucleotides close to the methylated nucleotide to be used as negative controls. These probes are then efficiently ligated by dNTP and SplintR ligase if the target adenosine is nonmethylated. Consequently, Taq DNA polymerase can efficiently amplify the ligated product upon the conversion of the ligated RNA into cDNA. On the other hand, the presence of a methyl moiety impairs the ligation efficiency, resulting in the amplification of relatively less PCR product. SELECT is a desirable choice for the analysis of site-specific single methylation marks on a target RNA. Despite its low cost and simplicity, it has low throughput and a high false-positive rate based on the efficiency of two distinct steps: elongation by Bst 2.0 DNA polymerase and ligation by SplintR ligase [123,124].

SELECT is a well-defined technique with two main steps: the single-base elongation activity and the nick ligation efficiency of DNA polymerases and ligases, respectively. Based on our existing experience, we suggest considering the following points in executing SELECT for site-specific assessment of methylation marks: (1) there should be a single nucleotide between the up and down probes; (2) diluting the enzymes in Diluent A facilitates their long-term storage, lowering costs as opposed to freshly preparing the enzymes each time as suggested by the manufacturers; (3) SplintR ligase in small quantities (0.5 unit) is sufficient in ligation reactions as opposed to higher amounts of T3 DNA ligase (12.5 unit); (4) the negative control, an unmethylated nucleotide near the desired methylated site, must be in the region in between −6 and +2 except for ±1. So, the up and down probes must be designed at one of these sites individually; (5) dTTP instead of dNTP appears to be slightly more efficient; and (6) it is important to design a qPCR adapter with a melting temperature above 50 °C at the two sides of the complementary strand of an RNA template.

## 4. Conclusions

A number of advances have been made in the field of RNA methylation, which has shifted its interest from the analysis of tRNA and rRNA to mRNA and other ncRNAs. In particular, the discovery of novel approaches that have enabled researchers to examine a variety of methylation at a site-specific or genome-wide manner has paved the way to uncover changes in the abundance of methylation marks in health and disease. Simultaneously, writers, erasers and readers of these biochemical modifications have been uncovered, which have been associated with numerous diseases. However, genome-wide approaches in particular can be applied to the analysis of a small fraction of biochemical modifications, necessitating the development of novel protocols. Along this line, third-generation sequencing technologies hold great promise as they do not require cDNA construction and permit direct detection of modifications with a much longer read. Additionally, existing sequencing-based approaches are still highly expensive, further requiring the development of protocols that reduce sequencing costs. Since each detection method has both advantages and limitations, the choice of method depends on the abundance of RNA modification, the type of RNA, the amount of starting material, cost, simplicity and the availability of proper infrastructure.

RNA methylation is one of the most dynamic research topics of recent times and clearly holds great promise for translation into clinic. Although it is a matter of debate whether this dynamic mechanism can be exploited to predict the progression of various diseases such as cancer, we believe that the identification of disease-specific methylation marks, e.g., RNA methylation signatures, should pave the way for its use in the clinic. Certainly, to facilitate this aim, cheaper and more convenient methods should be developed to speed up the research in this field. Alternatively, existing methods can be improved to lower analysis costs, leading to greater accessibility.
